# Bearded capuchin monkeys as a model for Alzheimer’s disease

**DOI:** 10.1038/s41598-024-56791-y

**Published:** 2024-03-15

**Authors:** Roberta Diehl Rodriguez, Maria Clotilde H. Tavares, Sonia Maria Dozzi Brucki, Leonel Tadao Takada, Maria Concepción Garcia Otaduy, Maria da Graça Morais Martin, Claudia Kimie Suemoto, Lea T. Grinberg, Claudia Costa Leite, Carlos Tomaz, Ricardo Nitrini

**Affiliations:** 1 Behavioral and Cognitive Neurology Group, Department of Neurology, University of São Paulo, 255 Dr. Enéas Carvalho de Aguiar, São Paulo, SP CEP 05403-000 Brazil; 2 Laboratório de Ressonância Magnética em Neurorradiologia (LIM-44) da Faculdade de Medicina da Universidade de São Paulo, 250 Dr. Enéas Carvalho de Aguiar, São Paulo, SP CEP 05403-000 Brazil; 3 Biobank for Aging Studies, University of São Paulo, 455 Dr. Arnaldo, São Paulo, SP CEP 01246-903 Brazil; 4 Laboratory of Neuroscience and Behavior, Department of Physiological Sciences, University of Brasília, Asa Norte, Brasília, DF CEP 70910-900 Brazil; 5 Primate Center, Institute of Biology, University of Brasília, Park Way-Núcleo Bandeirante, Brasília, DF CEP 71750-000 Brazil; 6 Memory and Aging Center, University of California San Francisco, San Francisco, CA 94158 USA; 7 Faculty of Medicine, Euro-American University Center-UNIEURO, Asa Sul, Brasilia, DF CEP 70297-400 Brazil

**Keywords:** Histocytochemistry, Proteins, Pathogenesis, Neurology, Dementia, Neurodegeneration, Neurodegenerative diseases

## Abstract

The absence of a natural animal model is one of the main challenges in Alzheimer’s disease research. Despite the challenges of using nonhuman primates in studies, these animals can bridge mouse models and humans, as nonhuman primates are phylogenetically closer to humans and can spontaneously develop AD-type pathology. The capuchin monkey, a New World primate, has recently attracted attention due to its skill in creating and using instruments. We analyzed one capuchin brain using structural 7 T MRI and performed a neuropathological evaluation of three animals. Alzheimer-type pathology was found in the two of the capuchins. Widespread β-amyloid pathology was observed, mainly in focal deposits with variable morphology and a high density of mature plaques. Notably, plaque-associated dystrophic neurites associated with disruption of axonal transport and early cytoskeletal alteration were frequently found. Unlike in other species of New World monkeys, cerebral arterial angiopathy was not the predominant form of β-amyloid pathology. Additionally, abnormal aggregates of hyperphosphorylated tau, resembling neurofibrillary pathology, were observed in the temporal and frontal cortex. Astrocyte hypertrophy surrounding plaques was found, suggesting a neuroinflammatory response. These findings indicate that aged capuchin monkeys can spontaneously develop Alzheimer-type pathology, indicating that they may be an advantageous animal model for research in Alzheimer’s disease.

## Introduction

Alzheimer’s disease (AD) is the main cause of dementia in humans and represents one of the greatest challenges for public health^1^. One significant obstacle in AD research is the absence of a natural animal model. A reliable animal model of disease is a mainstay of effective translational research^2^.

Humans and many mammals exhibit cognitive and behavioral declines associated with aging. Nevertheless, while cognitive decline in humans is mainly attributed to AD, aging has been assumed to cause the decline in other mammals^3^.

The presence of amyloid in the brains of older mammals has been reported; however, these animals have not been shown to exhibit neurofibrillary tangle (NFT) pathology^4,5^. Abnormally hyperphosphorylated tau has been reported in neurons in several aged mammalian models, but most of these animals lacked classic NFT^6,7^. The consensus that has prevailed for several years was that although amyloid plaques are present in many aged mammals, NFTs are absent, and therefore that AD is an exclusively human disease^8,9^.

The discovery of the amyloid precursor protein (APP) mutation made it possible to produce a transgenic mouse model of AD^2^. However, it was necessary to combine this mutation with other gene mutations to obtain plaque pathology, and even in this case, tau pathology and neurodegeneration are not observed^2^. Rosen et al. described NFT pathology and plaque-associated dystrophic neurites in a 41-year-old female chimpanzee^10^. Furthermore, Edler et al. analyzed the brains of 20 chimpanzees aged 37 to 62 years and observed Aβ in plaques and blood vessels and neurofibrillary pathology in the form of tau aggregates^11^. In contrast with the previous findings, these results suggest that “AD-like pathology is not limited to the human brain”^10,11^.

Chimpanzees could be regarded as the first identified natural animal model of AD. However, chimpanzees are at high risk of extinction, and prohibitive housing costs considerably limit their use in research^2^. More recently, hyperphosphorylated tau aggregates were found in mouse lemurs, and NFTs have been found in rhesus macaques and vervet monkeys^7,11–13^.

The capuchin monkey (*Sapajus* sp.) is one of South America's most common nonhuman primates. They inhabit Brazilian savanna-like environments and mangroves in the northeastern and central regions^14–16^. Sapajus exhibit sexual dimorphism, with males generally being larger (2.5–4 kg) than females (1.5–3 kg). This species features slower development, a larger size, a longer lifespan, and unique reproductive characteristics compared to neotropical primates. Female sexual maturity occurs at 3–4 years, and reproduction typically occurs after the individual reaches adult weight at 5 years^17^. Free-living males mature by the 7th–8th year, and those living in captivity can achieve mature earlier, and reproduction can continue until approximately 25 years of age^14,18^. The average lifespan of wild Sapajus is 34–36 years, while in captivity, they can live up to 55 years^19^.

The gyrification degree of the cerebral cortex is much greater in these primates than in other New World primates, and these primates also exhibit certain characteristics associated with hominids, including tool use, high encephalization, hand morphology, and dietary flexibility^15,16,18,20^.

Humans have the highest encephalization quotient (7.4–7.8) among primates^20^, followed by capuchins (4.8); the encephalization quotient is lower in chimpanzees (2.2–2.5) and gorillas (1.5–1.8)^20,21^.

Due to their complex cultural behavior and well-developed memory, capuchins are considered perhaps the most intelligent nonhuman primates in the Americas^14^. Foraging behavior includes locating, obtaining, processing, and eating food. The diet is based mainly on fruits (60%) but also includes other vegetables, insects, nectar, and even some oysters and crabs found in mangrove regions^14^.

Recent studies have shown that the bearded capuchin has extraordinary abilities^22–24^. Among them is stone tool production, which demonstrates “that the production of archeologically identifiable flakes and cores, as currently defined, is no longer unique to the human lineage”^23^. The wild bearded capuchin uses stone hammers and anvils to open hard-encapsulated food, fracture wood to access insects and larvae, and sticks as probes to access food, honey, and water^22^. In mangroves, they use wooden hammers and anvils to open crabs and mollusks^14^.

Captive and wild capuchins have been subjected to various cognitive tests, in addition to observations in a natural environment; these tests reveal diverse strengths in working memory, learning, delayed recall, executive function, and problem solving, including diverse skills depending on the place of origin and age of the monkey^25^. They have shown the ability to learn independently through exploratory tendencies as well as the ability to learn by observing older individuals^14,25–29^. In addition, they can exhibit behavior modifications behavior due to cohabitation with humans^30^.

Moreover, as elucidated by many authors^25,26,31^, capuchin monkeys, specifically *Sapajus* *libidinosus*, have demonstrated their cognitive potential through various behavioral tests. This includes cooperative behavior for reward acquisition, employing problem-solving strategies grounded in abstract rules, forming conceptual frameworks during task resolution, and demonstrating proficiency in short- and long-term operational memory tasks. These cognitive feats align capuchins with the cognitive capabilities observed in macaques (genus *Macaca*), underscoring the depth and breadth of their cognitive prowess^25,26^.

On the basis of all the information described above, particularly their demonstrated cognitive capabilities and brain morphology, this study aimed to investigate the occurrence of AD-type pathologies in capuchin monkeys.

## Results

**Figure 1:**
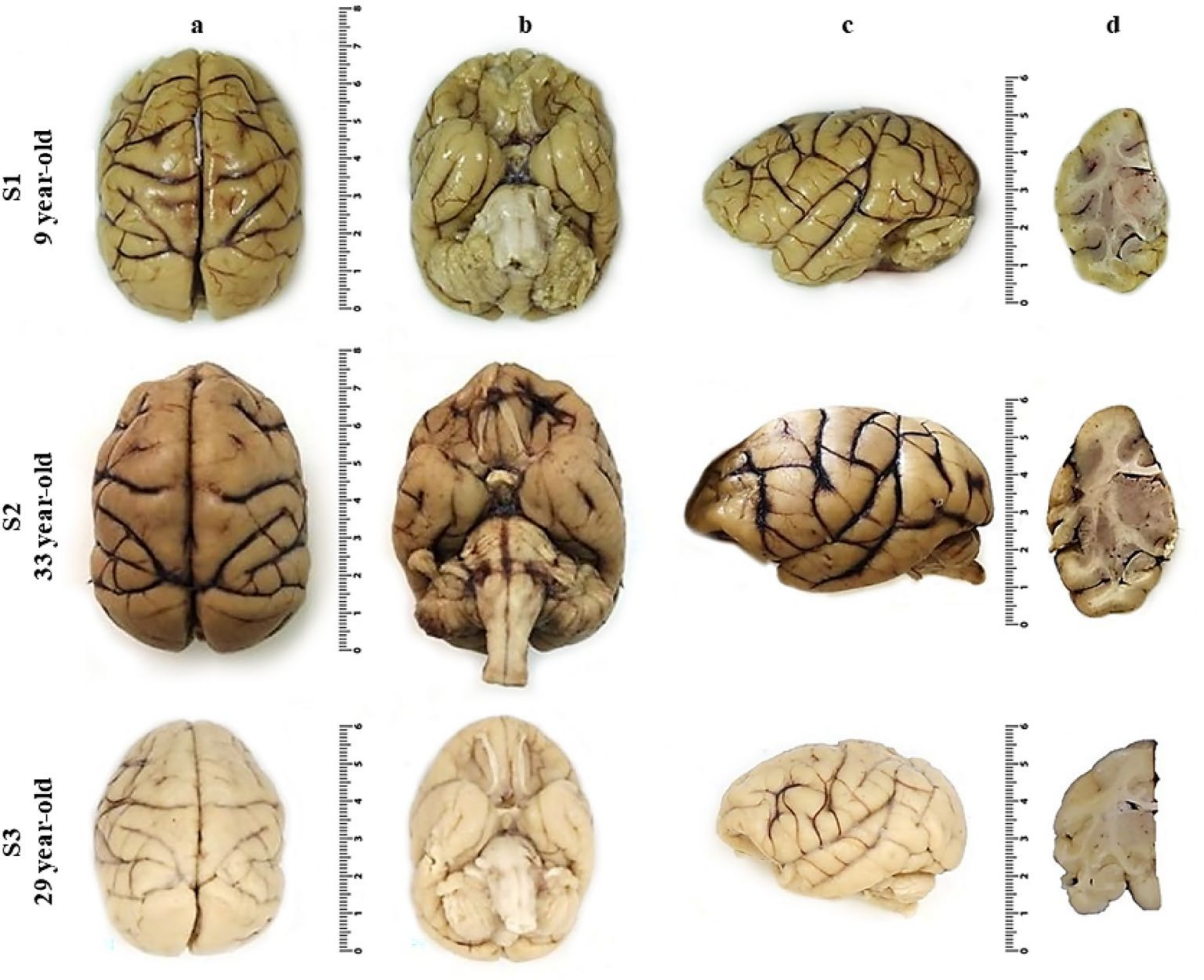
Macroscopic images. (**a**) Superior, (**b**) inferior, and (**c**) lateral views of the three capuchin monkey brains showing cortical gyrification. (**d**) Coronal section of the hippocampus at the level of the lateral geniculate nucleus. Scale unit: centimeters.

**Figure 2:**
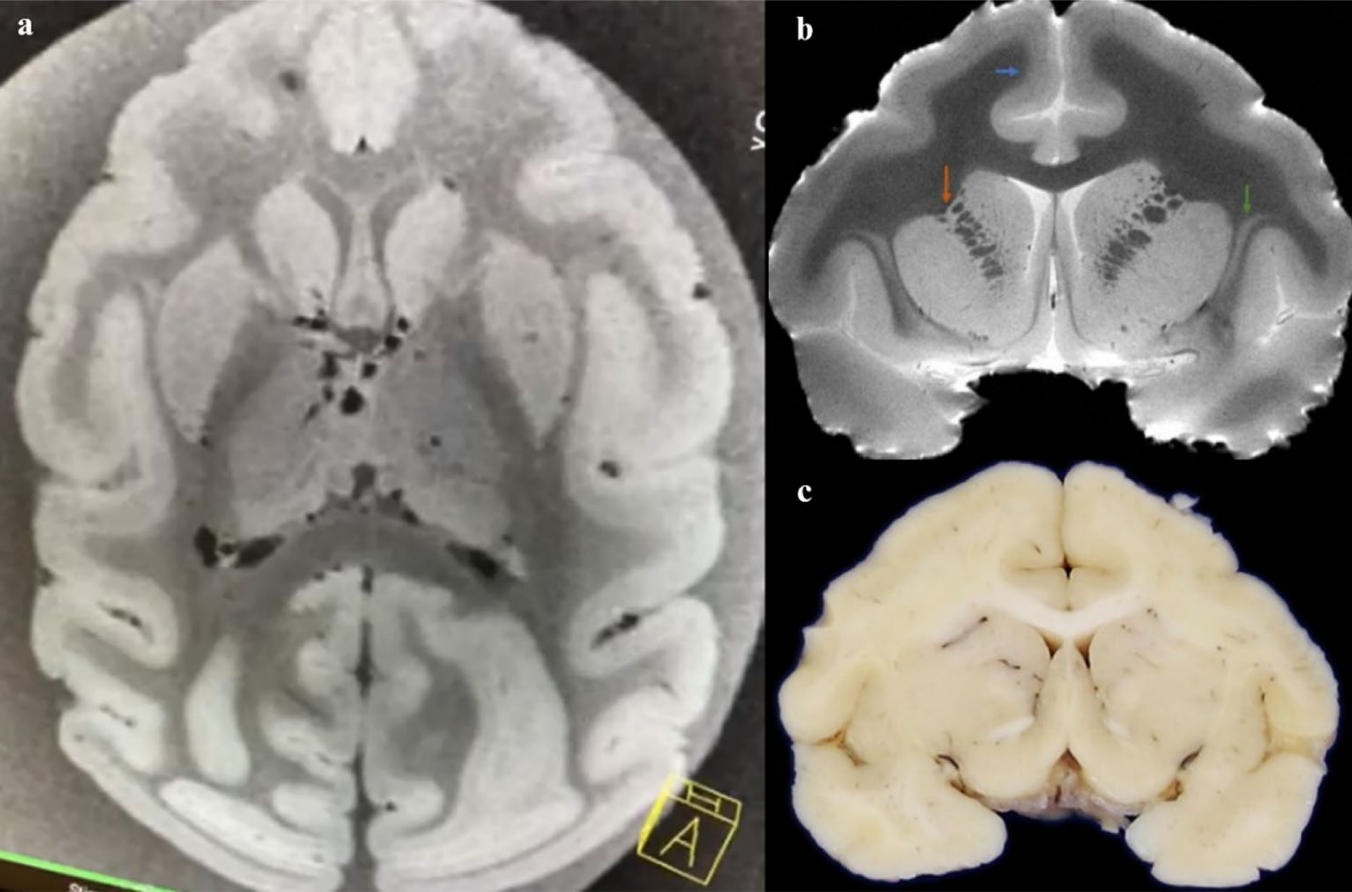
7 T ex situ brain magnetic resonance image (MRI) of a 29-year-old capuchin monkey. (**a**) Axial brain 7 T MRI image showing cortical gyrification. (**b**) In a T2-weighted image obtained from a 7 T MRI of a coronal slice, cortical lamination of the frontal cortex (blue arrow) and connections between the caudate and putamen (orange arrow) were observed. The claustrum can also be well identified (green arrow). (**c**) A macroscopic coronal slice at the same level as in (**b**).

Macroscopic evaluation of the capuchin monkey brain revealed considerable cortical gyrification (Fig. 1). In addition, a thick cortical ribbon was observed via MRI (Fig. 2).

### β-Amyloid deposits

AD-type pathology was observed in 29- and 33-year-old but not in 9-year-old capuchin. In both aged capuchin monkeys, β-amyloid (βA) deposits were observed in the hippocampus, amygdala, basal ganglia, and all cortical areas except the occipital cortex, where only amyloid angiopathy was found. A moderate density of diffuse and focal immunoreactive deposits was found. Although βA deposits were observed in both hemispheres, a greater burden was noted in the left hemisphere than in the right hemisphere in monkey S2.

**Figure 3:**
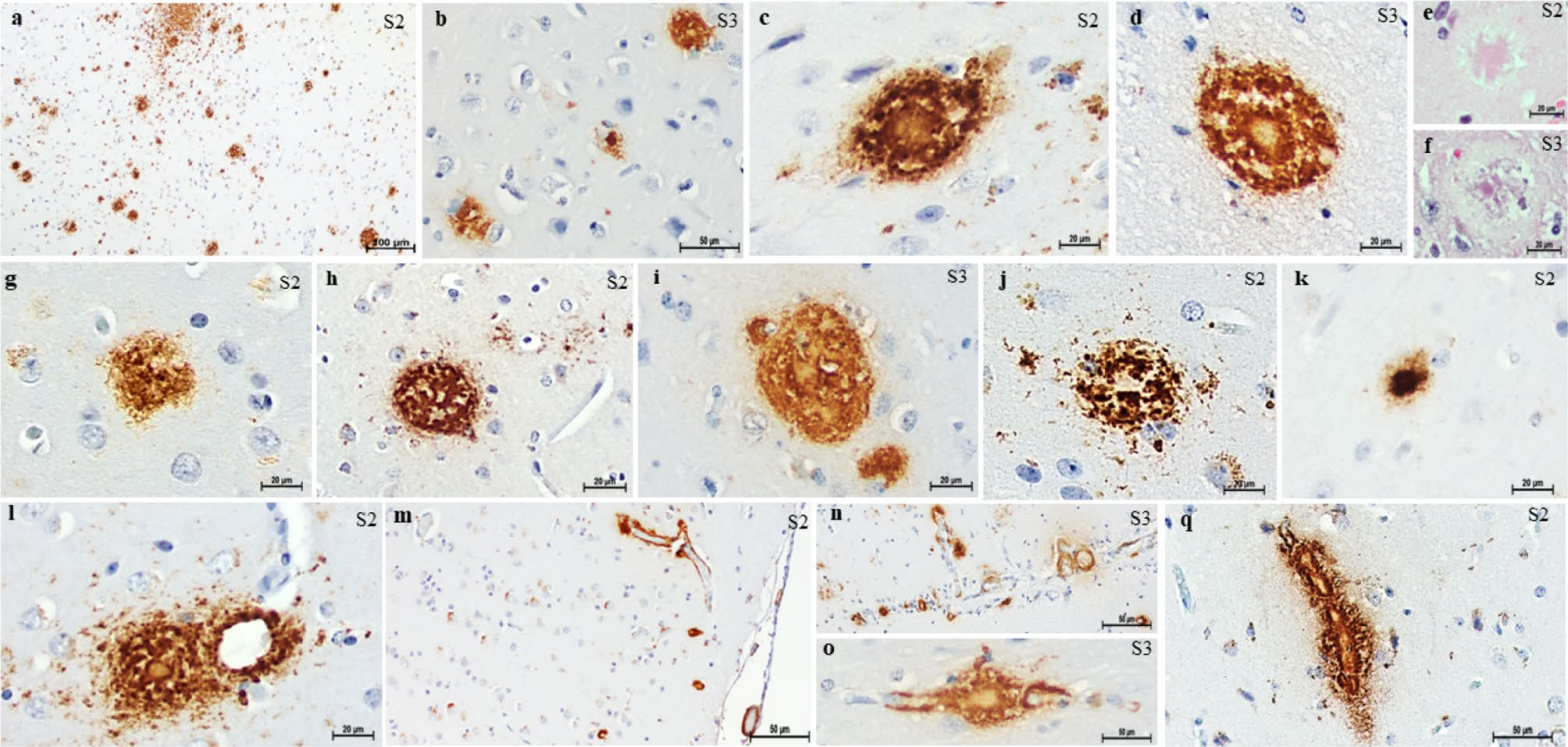
β-amyloid pathology was observed in the brains of 33-year-old (S2) and 29-year-old (S3) capuchins. Immunoreactive β-amyloid deposits in the frontal cortex (**a**) and temporal cortex (**b**). Plaques with amyloid cores (**c,d**) were also observed via hematoxylin–eosin staining (**e,f**). Focal β-amyloid deposits were observed as primitive (**g**) or mature plaques with different morphologies (**h–l**). β-Amyloid deposits in the meningeal and intracortical vessels were observed (**m,n**), and capillary involvement was observed (**o,q**). 4G8 antibody (**a–d,g–q**). Scale bars = 100 µm (**a**), 50 µm (**b,m,n,o,q**); 20 µm (**c–i**).

Widespread βA immunoreactivity was observed in the form of diffuse and focal deposits (Fig. 3). Most were focal, with a predominance of mature rather than primitive plaques (Fig. 3). Nevertheless, variable plaque morphology was observed, predominantly in the form of classical dense-cored plaques with a core-space-corona, coarse-grained plaque, burnt-out plaque, and juxtavascular plaque (Fig. 3). Plaques with amyloid cores were also observed via routine staining (Fig. 3).

In addition, in many cortical areas, βA deposits were found in the form of amyloid angiopathy in leptomeningeal and cortical vessels with involvement of not only arterioles but also capillaries (Fig. 3).

**Figure 4:**
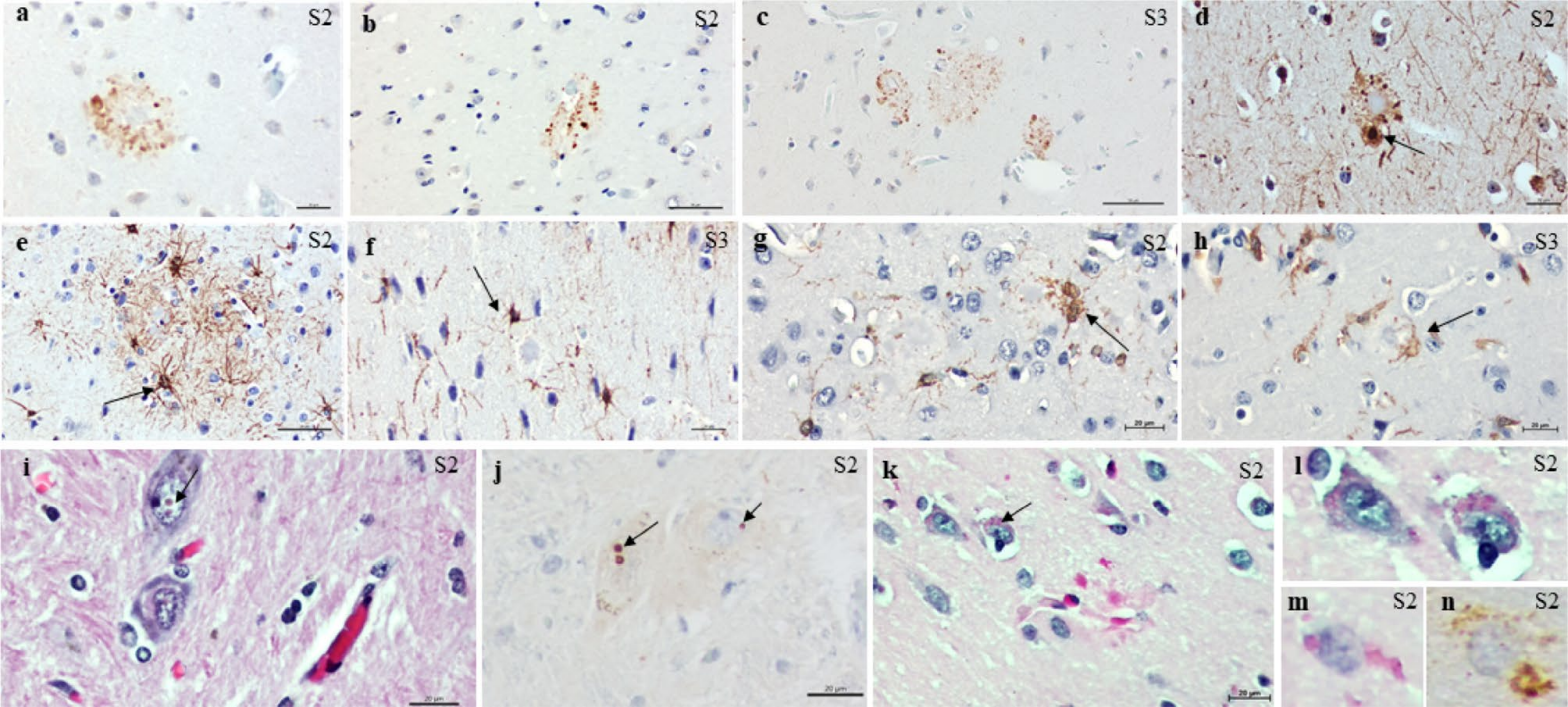
Dystrophic neurites, astrocytes, microglial changes, and age-related alterations were observed in capuchin monkeys. Abnormal neurites associated with plaques were identified via immunohistochemistry using antibodies against p62 (**a–c**) and neurofilament ((**d**); 2F11). In addition, immunohistochemical staining revealed hypertrophic astrocytes surrounding β-amyloid plaques ((**e,f**); GFAP) and microglial activation ((**g,h**); Iba1). Additionally, age-related alterations were observed via routine staining, specifically, (**i**) Marinesco bodies in neurons of the substantia nigra immunoreactive for the p62 antibody ((**j**); arrows) and (**k**) intracellular PAS-positive granular pigment (**k,m**). The PAS-positive pigment was β-amyloid; ((**n**); 4G8) immunoreactive. Scale bars: 20 µm  (**a,c,d,g–n**); 50 µm (**b,e,f**).

Additionally, plaque-associated neurites were identified using antibodies against p62 and neurofilaments (Fig. 4). In addition, hypertrophic astrocytes and microglial activation surrounding βA plaques were observed in both cases (Fig. 4)**.**

### Hyperphosphorylated tau deposits

**Figure 5:**
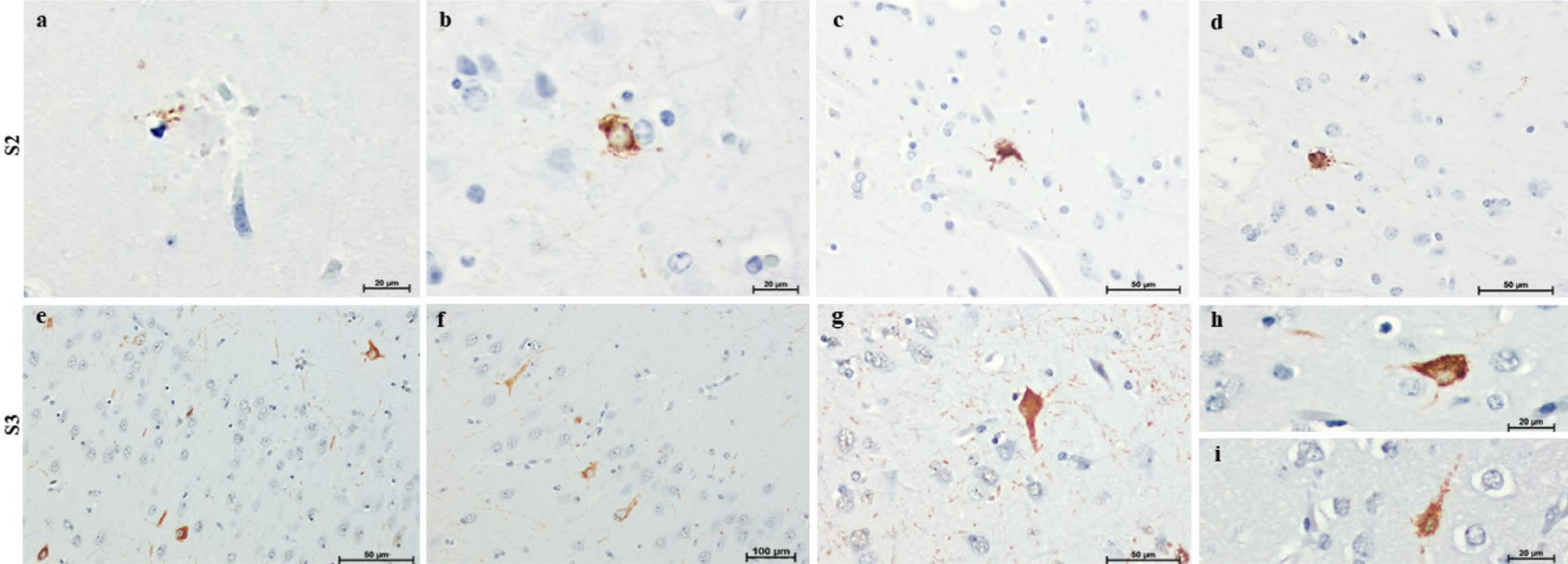
Hyperphosphorylated tau deposits are observed in the form of tau immunoreactive aggregates (AT8). (**a**) Surrounding the core of a classical neuritic plaque, (**b–i**) neurofibrillary pathology resembling pretangles and neurofibrillary tangles and neuropil threads. Scale bars: 20 µm (**a,b,h,i**); 50 µm (**c,d,e,g**), 100 µm (**f**).

Remarkably few dystrophic neurites were immunoreactive for hyperphosphorylated tau (*P*tau) in both cases (Fig. 5). In addition, in S2 sparse tau abnormal aggregates resembling pretangles, neurofibrillary tangles, and neuropil threads were found in the temporal and frontal cortexes using an AT8 antibody (Fig. 5). Higher burden of tau pathology was found in S3, mostly in the hippocampus (Fig. 5).

### Other neuropathological alterations

In addition to the presence of βA and Ptau aggregates, small intranuclear inclusions (Marinesco bodies) were observed in neurons of the substantia nigra (Fig. 4). These eosinophilic spherical nuclear inclusions were strongly immunoreactive for p62 (Fig. 4).

Additionally, a yellowish-brown Periodic acid–Schiff (PAS)-positive granular pigment, consistent with lipofuscin, was observed in the neuronal and glial cells via hematoxylin–eosin staining (Fig. 4). This PAS-positive pigment was positive for β-amyloid and p62.

Immunohistochemistry using TDP-43 and phosphorylated α-synuclein antibodies failed to reveal immunoreactive aggregates of these proteins in cortical, limbic, or brainstem regions.

## Discussion

AD-type pathology was found in two aged *Sapajus libidinosus* monkeys. Diffuse and focal βA deposits were observed in cortical and limbic areas. Notably, the deposits were usually more focal than diffuse. The density of mature plaques was greater than that of primitive plaques. These findings contrast with those of previous studies describing the predominance of diffuse deposits and primitive plaques in other New World monkeys, such as squirrel monkeys and marmosets^7,9,32^.

In addition, in our cases, neuritic plaques were frequently found in not only cortical but also subcortical areas such as the hippocampus, amygdala, and basal ganglia, contrasting with the predominance of diffuse deposits and primitive plaques described in squirrel monkeys and marmosets. Additionally, it is important to highlight the high density of mature plaques, even when compared to the density of cerebral amyloid angiopathy, and the presence of different morphologies similar to those observed in human brains in AD. Interestingly, cerebral amyloid angiopathy was not the predominant form of βA pathology in these two capuchin monkeys, unlike that observed in other New World species^7,9^.

In addition, abnormal aggregates of *P*tau were observed in hippocampus, temporal and frontal cortex, resembling neurofibrillary pathology. The Ptau immunoreactive pathology is very similar to that observed in the brains of human AD patients^33^.

The lack of Ptau in most dystrophic neurites surrounding classical neuritic plaques and the sparse density of neurofibrillary pathology can indicate the early stages of the disease^34^. However, these findings can be associated with species differences in the tau protein^6,35^.

Despite the low burden of Ptau pathology, the finding that these species can spontaneously develop AD-type pathology similar to that observed in humans is highly relevant. Additionally, the observed microglial activation and astrocyte hypertrophy indicate a neuroinflammatory response to AD-type pathology that can contribute to neurodegeneration or play a protective role^36^. Our findings identified a nonhuman primate species that may represent a new lower primate model appropriate for AD studies.

Additionally, studies of Marinesco bodies and lipofuscin accumulation have shown that they are aging-related microscopic findings, but their relationship with neurodegeneration is still unclear^37,38^. However, they are markers of cellular dysfunction and are associated with the main mechanisms for degrading proteins implicated in neurodegeneration, highlighting the value of the capuchin monkey as a natural model^37,38^.

The three animals participated in cognitive tests carried out at different times with satisfactory performance. However, in the present study, we obtained only scant information about cognitive decline or behavioral changes associated with aging in the two older capuchin monkeys. In future studies, we aim to obtain additional data to associate cognition and behavior with AD markers.

A literature review revealed that *Sapajus* sp. is a primate with many qualities that recommend its inclusion among AD animal models, with some advantages relative to others. Based on its genetic, behavioral, and morphological characteristics, the capuchin monkey is phylogenetically more closely related to humans than are other New World primates^19,39^.

Compared to Old World primates, capuchins are relatively small, as well as simpler and less expensive to maintain in captivity. *Sapajus libidinosus* surprised researchers with its skill in creating and using instruments^22,23^. It is a very curious and motivated animal that can be evaluated using neuropsychological tests similar to those used in other primates and humans^16,26–28^.

Compared with those of other primates, such as Saimiri^41^ and marmosets^2^, which have been proposed for use as animal models of AD, *Sapajus* has much more substantial cerebral cortex development and remarkable cognitive ability^14,16,21–23^. Moreover, the tau isoform expression pattern in marmosets may be more like that in mice than in humans^38^.

Using monkeys as natural models of AD is challenging due to the long time required for these animals to reach old age^2^. However, identifying cognitive decline and plasma biomarkers of βA and Ptau pathology in these animals may be highly relevant for developing evolutive biomarkers and new treatments for AD. It should also be considered that the Cebidae family, of which the bearded capuchin monkey is a member, is one of the less threatened primate families^40^. To conclude, *Sapajus libidinosus* should be considered an advantageous animal model for AD research.

## Materials and methods

This study has been approved by the local ethics committees. All animal care and experimental protocols were approved by the Ethics Committee for Animal Use of the University of Brasilia (SEI 23106.123230/2023-68). We did not perform any type of experiment relevant to this study during the lives of these primates. Management and maintenance to support the good welfare of these animals in captivity were carried out according to the Brazilian regulations for the Care and Use of Animals for Scientific Purposes established by the National Council for Control Animal Experimentation (CONCEA) (Lei Arouca 11.794/2008). The housing and maintenance conditions of the primates followed the laws and regulations of the Brazilian Institute of Environment and Renewable Natural Resources (IBAMA). Additionally, protocols and methods performed in the University of São Paulo were in accordance with Brazilian and international guidelines.

In 2016, a cooperative study was launched between the Department of Neurology at the University of São Paulo (USP) and the Primate Center at the University of Brasília (UnB) to investigate the presence of AD pathological markers in the brains of capuchin monkeys.

The Primate Center of the UnB aims to provide a captive breeding colony of Brazilian neotropical primates for ethological and biomedical research. This center is located on a farm of 4.340 ha (16030′′ S, 46030′′ W) in a protected area of an ecological reserve and houses primates in cages surrounded by nearby semideciduous tropical native gallery forests under natural light, temperature, and moisture conditions.

Upon arrival at the Primate Center, all monkeys undergo a thorough health assessment to verify their disease-free status, physical well-being, and readiness for integration. This entails a quarantine period, physical examination, weight and vital signs assessment, physical check-up, blood tests, fecal examination, and behavioral observation/socialization assessment. If anomalies are observed during a physical exam, supplementary tests such as X-ray or ultrasound may be performed. Postintegration, ongoing monitoring through regular health checks ensures the continual well-being of all individuals under the Primate Center's care.

The brains of three adult *Sapajus libidinosus* capuchin monkeys (2 males and 1 female) kept at the Primatology Center and who died of natural causes (the nine-year-old monkey died of pneumonia) were used for this study. The Brazilian Institute of the Environment and Renewable Natural Resources (IBAMA) collected the animals and sent them to the Primatology Center, where they arrived in adulthood. Therefore, the ages of the animals were estimated based on their morphological characteristics at the time of arrival plus the length of stay at the Primatology Center. The monkeys were kept in pairs in cages (4 m long × 2.9 m wide × 2 m high) with two concrete walls separating adjacent cages and wire mesh as the front, back, and roof, forming an external/semi-internal housing system. Each cage contained a suspended wooden nest box, several wooden perches at different heights, a food tray where the animals were fed, and a thick layer of natural dry leaves on the floor. The animals had olfactory and acoustic contact but not visual contact with other colony members. Fresh fruits and vegetables were provided daily from 7:30 a.m. until 5:00 p.m. Primate food and fresh water were provided ad libitum. The animals received ongoing veterinary monitoring and underwent clinical evaluations and monthly weighings.

A complete neuropathological evaluation was performed for the three animals in the study (9- and 33-year-old males and a 29-year-old female). Moreover, a structural 7 T magnetic resonance image (7 T MRI) was acquired from only one patient (a 29-year-old female) before and after brain slicing (Table 1).

**Table 1:** Monkeys characteristics.

Identification	Sex	Age at death (years)^a^	7 T MRI^b^	AD-type of pathology
S1	Male	9 (adult)	No	No
S2	Male	33 (aged capuchin)	No	Yes
S3	Female	29 (aged capuchin)	Yes	Yes

^a^Estimated age.

^b^Postmortem.

An experienced veterinarian performed the brain extractions from the monkeys after their deaths. Brain tissue was placed in 4% buffered paraformaldehyde within 12 h of death and fixed for three weeks. Consecutive coronal sections from the fixed brain were embedded in paraffin, and 5 μm sections from paraffin blocks were used for staining and immunohistochemistry evaluation. All brain sections were stained with hematoxylin and eosin.

Immunohistochemistry was performed with antibodies against β-amyloid (4G8, 1:10.000; BioLegend), tau phosphorylated at Ser^199–202^-Thr^205^ (AT8, 1:400; Thermo Fisher), p62 LCK ligand (p62, 1:500; BD Bioscience), phosphorylated transactivation response DNA-binding protein of 43 kDa (TDP-43, 1:500; BioLegend), α-synuclein phosphorylated at Ser^129^ (81A, 1:500; BioLegend), 68 kDa neurofilament (2F11; Sigma), glial fibrillary acidic protein (GFAP; Dako) and microglia markers (Iba1; E5N4J, Cell Signaling) in selected sections of both hemispheres to analyze the following areas: frontal cortex, temporal cortex, parietal cortex, occipital cortex, anterior cingulate cortex, hippocampus, amygdala, basal ganglia, thalamus, mesencephalon, pons, medulla oblongata, and cerebellum.

### Statement on animal research

#### Study design

The present study is a prospective descriptive study. Our aim was to investigate the presence of neuropathological changes characteristic of Alzheimer’s disease in capuchin monkeys who died of natural causes. We did not perform any type of experiment relevant to this study during the lives of these primates.

#### Sample size

Three adult *Sapajus libidinosus* capuchin monkeys (2 males and 1 female) kept at the Primatology Center and who died of natural causes were included in this study. The Primatology Center of the University of Brasilia is located on a farm of 4.340 ha (16030" S, 46030" W) in a protected area of an ecological reserve and houses primates in cages surrounded by nearby semideciduous tropical native gallery forests under natural light, temperature, and moisture conditions. The main objective of the center is to provide a captive breeding colony of Brazilian neotropical primates for ethological and biomedical research.

Animals arrive at the Primatology Center in adulthood. Therefore, the ages of the animals were estimated based on their morphological characteristics at the time of arrival, plus the length of their stay at the Primatology Center. The animals were kept in pairs in cages (4 m long × 2.9 m wide × 2 m high), which consisted of two concrete walls separating adjacent cages and wire mesh at the front, back, and roof, forming an external/semi-internal housing system. Fresh fruits and vegetables were provided daily from 7:30 a.m. until 5:00 p.m. The animals were subjected to ongoing veterinary monitoring and clinical evaluation and monthly weighing.

#### Inclusion criteria

*Sapajus libidinosus* at the Primatology Center of the University of Brasilia who died from natural causes.

#### Exclusion criteria

Animals not sampled within 24 h postmortem.

#### Randomization

Not applicable.

#### Blinding

Not applicable.

#### Outcome measures

Not applicable.

#### Experimental animals

Not applicable.

#### Experimental procedures

Not applicable. (Neuroimaging and neuropathological studies were carried out after death of natural causes.)

#### Results

We described the presence of Alzheimer’s type pathology in the two aged capuchin monkeys (29- and 33-year-old monkeys).

## Data Availability

All the data are available in the main text.
